# Degenerative injuries of the metatarsophalangeal plantar plate on magnetic resonance imaging: a new perspective

**DOI:** 10.31744/einstein_journal/2022AO6543

**Published:** 2022-04-07

**Authors:** Tania Szejnfeld Mann, Caio Augusto de Souza Nery, Daniel Baumfeld, Eloy de Ávila Fernandes

**Affiliations:** 1 Hospital Israelita Albert Einstein São Paulo SP Brazil Hospital Israelita Albert Einstein, São Paulo, SP, Brazil.; 2 Universidade Federal de Minas Gerais Belo Horizonte MG Brazil Universidade Federal de Minas Gerais, Belo Horizonte, MG, Brazil.; 3 Escola Paulista de Medicina Universidade Federal de São Paulo São Paulo SP Brazil Escola Paulista de Medicina, Universidade Federal de São Paulo, São Paulo, SP, Brazil.

**Keywords:** Metatarsalgia, Magnetic resonance imaging, Plantar plate, Joint instability, Rupture, Metatarsophalangeal joint

## Abstract

**Objective:**

The magnetic resonance imaging diagnostic criteria for a complete tear of metatarsophalangeal plantar plate are well-established. However, more subtle abnormalities can also occur and be a source of pain. The objective of this study is to determine the prevalence of degenerative plantar plate injuries in patients with metatarsalgia who underwent forefoot magnetic resonance imaging and describe the main abnormalities found. The hypothesis is that mild capsular fibrosis will have high sensitivity but low specificity for plantar plate degenerative injuries.

**Methods:**

A retrospective cross-sectional study was conducted with 85 patients (105 feet) with metatarsalgia who underwent forefoot magnetic resonance imaging using a specific protocol to study metatarsophalangeal plantar plate. The experiment observer classified second toe plantar plate as normal, complete rupture or degenerative lesion and described the main magnetic resonance imaging findings.

**Results:**

A normal plantar plate was observed in 75 (71.4%) of the 105 feet assessed, in 25 (24%) feet there were degenerative plantar lesions, and in 5 (4.6%) feet there were complete ruptures. Degenerative injury of the plantar plate was best identified in coronal short axis intermediate-weighted images, with high sensitivity (92%). Pericapsular fibrosis below the intermetatarsal ligament was identified in 96% of cases, with high sensitivity (96%) for diagnosis of degenerative plantar plate injury.

**Conclusion:**

Degenerative lesions of the metatarsophalangeal plantar plate were more prevalent than complete ruptures and were best viewed in coronal short axis intermediate-weighted sequences. Pericapsular fibrosis below the intermetatarsal ligament was the indirect finding most strongly associated with degenerative plantar plate injury.

## INTRODUCTION

Metatarsalgia is one of the most common complaints of patients who have problems with their feet.^(1)^ There is a wide variety of causative factors, but all of them appear to be related to the mechanics of walking, anatomy, and/or deformities of the foot and ankle.^(1)^Recently, interest has been increasing in one of the differential diagnosis possibilities: metatarsophalangeal (MTP) plantar plate injuries. Rupture of the plantar plate is characterized by MTP pain in the lateral rays (II to V), especially in the second joint.^(2,3)^ The plantar plate is the main ligament responsible for sagittal stability of the lesser toes.^(4)^ During the acute phase, injuries to this collagen structure present pain and edema over the metatarsal head.^(5)^ However, 93% of cases progress gradually, without sudden onset, and almost 70% of patients only seek care after six months of symptoms.^(2)^ Since MTP instability is frequently underdiagnosed, many patients go untreated and symptoms of deformity can progress. With time, the plantar plate can become attenuated and, eventually, develop a tear that causes instability and deformity of the joint. Widening of the interdigital space and a positive MTP “drawer” test^(6)^ are later manifestations.^(7-9)^ Nery et al.,^(7)^ consider that grade 0 and 1 instability are earlier stages of this disease, and that magnetic resonance imaging (MRI) is the most widely used technique for early and/or late identification of these injuries.

Magnetic resonance imaging is considered the most sensitive and specific test^(10,11)^for diagnosis of metatarsophalangeal plantar plate (MTP-PP) injuries. Yao et al.^(12)^ described diagnostic criteria, and efforts have since been made to reproduce and improve them. Other authors^(13)^ have described findings that confirm diagnosis, such as presence of pericapsular fibrosis, which is present in 98% of cases. The MRI diagnostic criteria for normal plantar plate and complete tear are well-established.^(12,14)^ Degenerative plantar plate injuries are frequent injuries, often underestimated or undervalued in studies, which constitute a clinical scenario that should not be disregarded as a cause of metatarsalgia.

## OBJECTIVE

To determine the prevalence of degenerative plantar plate injuries in patients with metatarsalgia who underwent forefoot magnetic resonance imaging and describe the main abnormalities found. The hypothesis of the present study is that mild capsular fibrosis will have high sensitivity but low specificity for plantar plate degenerative injuries.

## METHODS

### Patients

This retrospective cross-sectional study included 110 forefoot MRIs without contrast in a series of 85 consecutive patients (105 feet) with metatarsalgia obtained at one institution between January 2017 and January 2018. Only patients older than 18 were included. Subjects with a history of diabetes, inflammatory diseases, or previous surgery were excluded from the analysis. Images were anonymized (TSM) and reviewed by a musculoskeletal radiologist (EAF) with more than 20 years of experience, who evaluated them blindly at two different times (six-month interval), to enable determination of intraobserver agreement. The study was approved by the Ethics Committee of the *Universidade Federal de São Paulo* (UNIFESP), registration #1.794.602, CAAE: 60480216.1.1001.5505. Patients were not asked to sign a consent form because of the retrospective nature of this study. The STROBE (Strengthening the Reporting of Observational Studies in Epidemiology) checklist for cross-sectional studies was used to collect and describe the data.^(15)^

### Ethical approval

All procedures performed in studies involving human participants were in accordance with the ethical standards of the institutional and/or national research committee and with the 1964 Helsinki declaration and its later amendments or comparable ethical standards.

### Imaging techniques

Magnetic resonance imaging was obtained using a 1.5 T scanner (Essenza, Siemens Medical Systems, Erlangen, Germany) or a 3.0 T scanner (Verio, Medical Systems, Erlangen, Germany) using a dedicated extremities coil. Images were acquired with patients in prone position with the feet in plantar flexion, as this results in less magic angle effect, less patient movement, and slight plantar shift of interdigital soft tissue. The standard protocol consisted of T1-weighted spin-echo images and intermediate-weighted turbo spin-echo (TSE) images with fat suppression in the long axis, short axis, and sagittal plane (Table 1). The long axis was aligned parallel to the heads of the second to fourth metatarsals, the short axis was aligned parallel to the tarsometatarsal joint, and the sagittal plane was aligned parallel to the axis of the second metatarsus.

**Table 1:** Magnetic resonance imaging parameters for all sequences (1.5 T and 3.0 T protocols)

Parameters	Short axis		Sagittal plane		Long axis	
		
	T1-weighted SE sequence	Intermediate-weighted SE sequence	T1-weighted SE sequence	Intermediate-weighted SE sequence	T1- weighted SE sequence	Intermediate-weighted SE sequence
TR (ms)	459-774	1,800-6,040	414-805	2,930-3,080	414-721	2,200-3,470
TE (ms)	12	43-46	12-15	38-51	11-12	38-50
FOV (mm)	110-140	110-140	140-150	120-140	130-140	130-140
Slice thickness/gap (mm)	3.0/0	3.0/0	3.0/0	3.0/0	2.0-3.0/0	2.0-3.0/0
No of slices	24-30	24-30	20-24	20-24	16-18	16-18
Matrix	184-187 x 75-384	320-384 x 70-75	187-448 x 70-384	182-320 x 75-320	187-448 x 70-384	182-320 x 80-320

SE: spin-echo; TR: repetition time; TE: echo time; FOV: field of view.

### Qualitative analysis

Images were retrospectively reviewed by the experiment observer using established MRI criteria for plantar plate tear,^(12)^ classifying 2^nd^MTP-PP images as normal, degenerative, or ruptured, as follows:

Normal plantar plate: a uniform low-intensity signal below the metatarsal head in the sagittal plane in intermediate-weighted and T2-weighted fat-suppressed sequences. On the short axis, the plantar plate appears as a U-shaped band of low-intensity signal, centralized over the head of the metatarsal.^(12)^ A zone of high-intensity signal in the mid region of the phalangeal attachment of the plantar plate measuring up to 2.5mm was also considered normal, because it constitutes an anatomic recess.^(16)^

**Figure 1:**
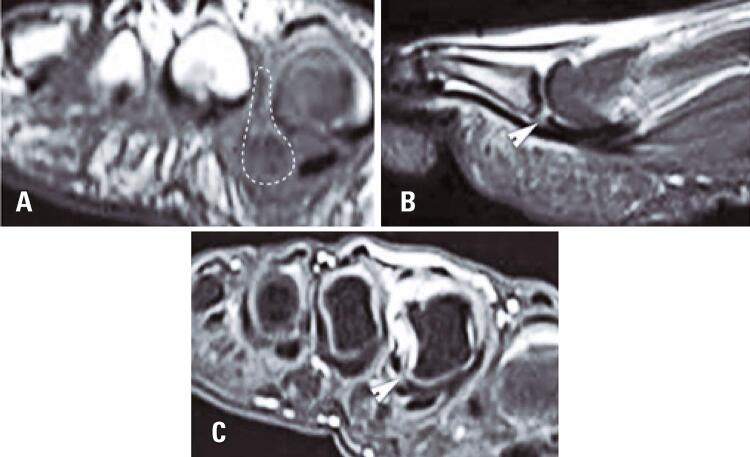
Sixty-two-year-old woman with complete plantar plate tear of the second toe. (A) short axis T1-weighted image with intermediate signal soft tissue (dotted line bordering) adjacent to the plantar plate of the second metatarsophalangeal joint and extends above the intermetatarsal ligament; (B) sagittal T2-weighted with suppression of fat sign image showing classic direct sign of plantar plate rupture with focal hypersignal separating the free margin of the plantar plate and the base of the proximal phalanx measuring 4mm (arrow);(C) Short axis T2-weighted with suppression of fat sign image showing focal discontinuity of the lateral margin of the 2nd plantar plate (triangle)

Complete plantar plate rupture: a high-intensity signal in T2-weighted images at the attachment of the plantar plate is accepted as a direct sign of rupture^(12)^(Figure 1). These injuries appear as high-intensity signals in intermediate-weighted and T2-weighted images with fat suppression. Retraction of the plantar plate is best assessed in the sagittal plane.

**Figure 2:**
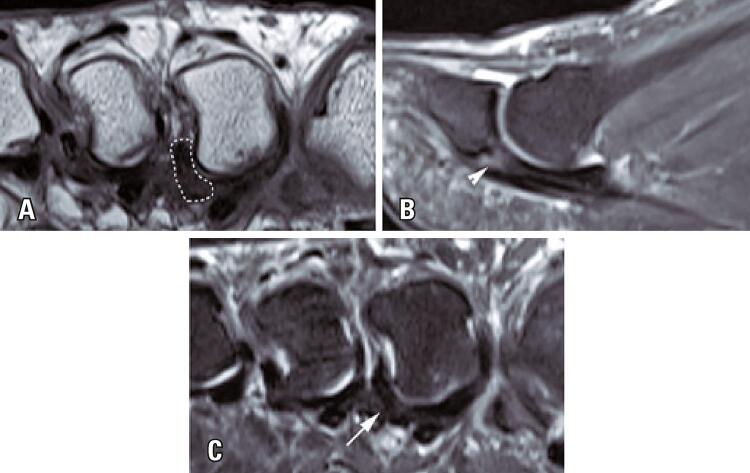
Fifty-four-year-old woman with degenerative second toe plantar plate injury. (A) short axis T1-weighted image illustrating eccentric thickening of pericapsular soft tissue below the intermetatarsal ligament, indicated by the broken line bordering the lateral capsule of the second MTP; (B) sagittal T2-weighted with suppression of the fat sign image showing a small plantar hypersignal (arrowhead) permeating the homogenous plantar plate hyposignal; (C) short axis intermediate-weighted image showing heterogeneous thickening in the lateral margin of the plantar plate (arrow)

Degenerative injury: presence of an intermediate signal permeating the plate, with no abrupt breakdown, or thickening or thinning of the plantar plate in the short axis and the sagittal plane, in intermediate-weighted images with suppression of the fat signal (Figure 2).

Signs defined as indirect indications of plantar plate injury, such as subcortical bone marrow edema, signal intensity at the base of the proximal phalanx, flexors tenosynovitis, joint effusion, intermetatarsal bursitis, and pericapsular fibrosis^(13)^ (thickening of soft tissues adjacent to the joint capsule, with intermediate signal in T1 and T2-weighted with fat suppression sequences) were noted. Pericapsular fibrosis was classified as minor, when restricted to the lower 1/3 of the metatarsal head, below the intermetatarsal ligament, and extensive, when observed along the entire lateral margin of the metatarsal head, extending above the intermetatarsal ligament. Presence of interdigital neuroma in the 2^nd^ and 3^rd^ spaces was also noted.

### Statistical analysis

Parametric statistical tests were used, because the normality of primary outcome quantitative variables was assessed using the Kolmogorov-Smirnov test and distributions were considered normal. Student’s *t* test was used to determine whether there were statistical differences between means. Sensitivity, specificity, accuracy, positive predictive values, and negative predictive values were calculated for the findings described. Intraobserver agreement was analyzed using the Kappa agreement index. A 5% cutoff for rejection of the null hypothesis was adopted to set the level of statistical significance (p<0.05). Statistical Package for the Social Sciences (SPSS) version 20, Minitab 16, and Microsoft Excel 2010 were used for these statistical analyses.

## RESULTS

**Figure 3:**
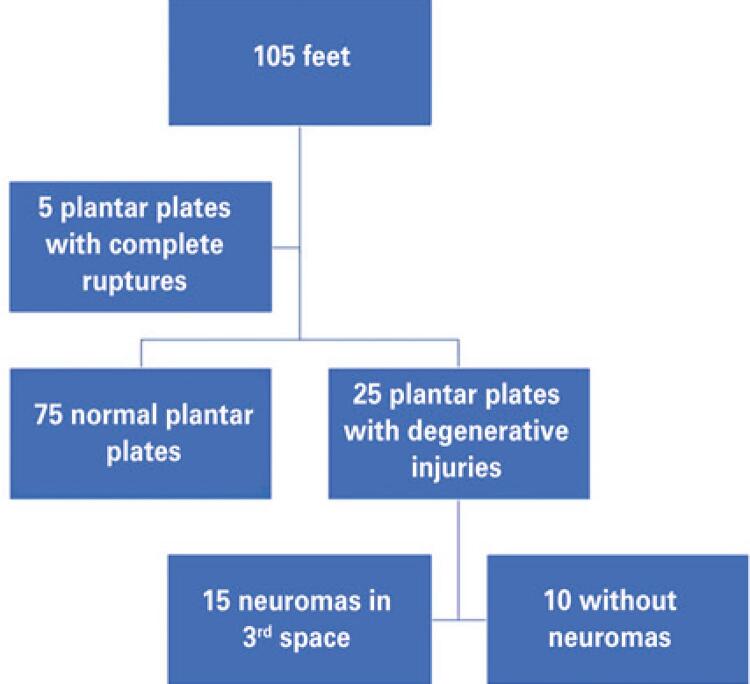
Images analyzed in the study

Figure 3 contains a flowchart illustrating classification of the images analyzed in the study. Five studies were excluded because the three patients presented inflammatory disease and two patients presented previous surgery (correction of hallux valgus deformity). A total of 75 of the 105 plantar plate images analyzed were classified as normal, 30 cases were classified as having a plate injury, 25 with degenerative injuries, and five with complete ruptures. Fifteen of the degenerative plantar plate injuries of the 2^nd^ metatarsal also had neuromas of the 3^rd^interdigital space. Table 2 shows the clinical characteristics of the cases included in the study, classified as normal plantar plate or degenerative injury. There was a predominance of females (75%) in both groups. Mean age was greater in the group with degenerative injuries than in the normal group.

**Table 2:** Clinical characteristics of cases classified as normal plantar plate and degenerative injury

Classification	Sex	Age (mean)	Standard deviation	p value
Normal	47F/15M	43.5	14.6	<0.001
Degenerative	17F/6M	56.3	12.3	

F: female; M: male.

Table 3 shows the indirect signs of injury observed in the subsets with and without plantar plate injuries. The prevalence of pericapsular fibrosis below the ligament was significantly higher among those with degenerative injuries (p<0.001). Proximal phalanx enthesitis was observed in 8% of cases with degenerative injury and was not observed in any of the cases with normal plantar plate. Pericapsular fibrosis below the ligament demonstrated 96% sensitivity and 61% specificity for diagnosis of degenerative plantar plate injuries and had a negative predictive value of 97.9%. Presence of pericapsular fibrosis above the intermetatarsal ligament had high specificity, but low sensitivity (Table 4). Only three studies presented interdigital neuroma of the second space. In all these studies, the plantar plate was classified as normal. Four feet presented concomitant bursitis of the second space and plantar plate lesion (three degenerative lesions and one complete rupture).

**Table 3:** Indirect findings in forefeet MRI of patients with metatarsalgia, classified into subsets with degenerative plantar plate injuries and normal plantar plate

Indirect signs of PP injury in MRI	Indirect MRI features of PP tears	Degenerative	Normal	Total	p value

		n (%)	n (%)	n (%)	
Extensive pericapsular fibrosis	Yes	1 (4.0)	0 (0)	1 (1.0)	0.082
	No	24 (96.0)	75 (100)	99 (99.0)	
Minor pericapsular fibrosis	Yes	24 (96.0)	29 (38.7)	53 (53.0)	<0.001
	No	1 (4.0)	46 (61.3)	47 (47.0)	
Enthesitis, 2^nd^ toe proximal phalanx	Yes	2 (8.0)	0 (0)	2 (2.0)	0.013
	No	23 (92.0)	75 (100)	98 (98.0)	
Flexors tenosynovitis, 2^nd^ MTP	Yes	1 (4.0)	1 (1.3)	2 (2.0)	0.409
	No	24 (96.0)	74 (98.7)	98 (98.0)	
Effusion, 2^nd^ MTP	Yes	12 (48.0)	25 (33.3)	37 (37.0)	0.188
	No	13 (52.0)	50 (66.7)	63 (63.0)	
Bursitis, 2^nd^ space	Yes	4 (16.0)	14 (18.7)	18 (18.0)	0.764
	No	21 (84.0)	61 (81.3)	82 (82.0)	
Bursitis, 3^rd^ space	Yes	7 (28.0)	34 (45.3)	41 (41.0)	0.127
	No	18 (72.0)	41 (54.7)	59 (59.0)	

MRI: magnetic resonance imaging; MTP: metatarsophalangeal; PP: plantar plate.

**Table 4:** Sensitivity, specificity, accuracy, positive predictive value, and negative predictive value of indirect signs of degenerative injury

Predictive values of indirect signs of plantar plate injury	Accuracy (%)	Sensitivity (%)	Specificity (%)	PPV (%)	NPV (%)
Extensive pericapsular fibrosis	76.0	4.0	100	100	75.8
Minor pericapsular fibrosis	70.0	96.0	61.3	45.3	97.9
Enthesitis, 2^nd^ toe	77.0	8.0	100	100	76.5
Tenosynovitis of 2^nd^ toe flexors	75.0	4.0	98.7	50.0	75.5
Effusion, 2^nd^ MTP	62.0	48.0	66.7	32.4	79.4
Bursitis, 2^nd^ space	65.0	16.0	81.3	22.2	74.4
Bursitis, 3^rd^ space	48.0	28.0	54.7	17.1	69.5

PPV: positive predictive value; NPV: negative predictive value; MTP: metatarsophalangeal.

In the intraobserver analysis, agreement was almost perfect, with a Kappa index of 0.873.

Direct findings of degenerative injury were best evaluated on short axis intermediate-weighted images. Sensitivity was 92%, specificity was 61%, and the negative predictive value was 95.8% (Table 5).

**Table 5:** Sensitivity, specificity, accuracy, positive predictive value, and negative predictive value of magnetic resonance imaging sequences for assessment of degenerative metatarsophalangeal plantar plate injury

MRI sequences for assessment of degenerative plantar plate injury	Accuracy (%)	Sensitivity (%)	Specificity (%)	PPV (%)	NPV (%)
Degenerative short axis intermediate-weighted fat saturation	69.0	92.0	61.3	44.2	95.8
Degenerative short axis T1-weighted fat saturation	68.0	84.0	62.7	42.9	92.2
Degenerative sagittal intermediate-weighted fat saturation	73.0	80.0	70.7	47.6	91.4

PPV: positive predictive value; NPV: negative predictive value; MRI: magnetic resonance imaging; MTP-PP: metatarsophalangeal plantar plate.

## DISCUSSION

Adopting the criteria for degenerative plantar plate injuries proposed by Yao et al.,^(12)^ we identified 24% of degenerative plantar plate injuries of the 2^nd^ MTP in our sample and just 5% with classic signs of complete rupture. Umans et al.,^(17)^ retrospectively analyzed images of 100 forefoot MRIs from patients with metatarsalgia, using similar methodology to this study, finding that 40% had a complete injury of the plantar plate of the 2^nd^ or 3^rd^ MTP, although other authors^(18,19)^ found prevalence rates of around 20%. None of these authors specifically described more subtle abnormalities, suggestive of degeneration of the plantar plate. Linklater et al.^(14)^ describe in detail and illustrate the MRI findings in degenerative MTP-PP injuries, but do not report their prevalence or importance. It is interesting to note that while some authors identify more subtle changes to the plantar plate,^(12,14,18,20)^ so far, no studies have evaluated the prevalence of degenerative plantar plate injuries or described their main characteristics. In fact, degenerative plantar plate injuries could be included in the differential diagnosis of forefoot pain, constituting a more insidious stage, prior to complete plantar plate rupture.

During initial deterioration of the plantar plate, there are no digital deformities and joint instability may be absent.^(8,9)^ As the condition progresses, separation of the toes may occur, with eventual progression to multi-plane deformities. Mendicino et al.^(8)^ assessed MRI images from patients with pain under the metatarsal head, without joint dislocation, and found similar changes to those described as degenerative plantar plate injuries in our article. They referred to these findings as MTP predislocation syndrome.

The images analyzed in this study and classified as degenerative plantar plate injuries probably correspond to those that clinically present grade 0 or 1 MTP instability, as suggested by Nery et al.^(11)^The MTP “drawer” test^(6)^ was not performed in this study.

Sixty percent of the degenerative injury cases had neuroma of the 3^rd^interdigital space. Neuroma of the 3^rd^ space is often found in association with plantar plate injuries.^(13)^In general, resection of 3^rd^ space neuromas is followed by a high rate of satisfaction.^(21)^However, Coughlin et al.^(21)^ warn that plantar plate involvement could be one of the causes of dissatisfaction after surgical resection. Their best results were observed in subsets that only had neuromas or in which MTP instability was also repaired. Patients who had milder plantar plate injuries and only underwent resection of neuromas had the least satisfactory results. These findings confirm the need to identify the entire spectrum of MTP-PP changes, even the most discrete ones.

As expected, degenerative plantar plate injuries were most common among women over the age of 50, as has been observed by other authors in patients with plantar plate ruptures.^(3)^

Another relevant aspect was the way in which we categorized pericapsular fibrosis. In cases with degenerative plantar plate injuries, fibrosis was limited to the lower third of the metatarsal head, below the intermetatarsal ligament, in contrast with the complete rupture cases, in which fibrosis extended along the entire lateral margin of the metatarsal head. This finding was relevant because it was present in 96% of cases. Other authors have also identified the importance of pericapsular fibrosis as an indirect finding indicative of plantar plate rupture.^(16,20)^ Other indirect signs associated with complete rupture, such as joint effusion, enthesitis, and tenosynovitis of flexor tendons were not statistically significant.^(20)^ These data support the hypothesis that degenerative injury is part of a subset of plantar plate injuries, with a more chronic clinical course. Regarding the possibility of confusion in classification of pericapsular fibrosis with the interdigital neuroma, this study did not identify any concomitant case of neuroma in the second space with the plantar plate lesion. Possibly, the examiner’s experience helped in this diagnosis.

Normal plantar plate is most effectively analyzed in intermediate-weighted and T2-weighted fat-suppressed sequences, in the short axis, the plantar plate appears as a C-shaped low-signal-intensity band centered under the metatarsal head. A bright T2 signal defect at the insertion of the plantar plate observed in intermediate-weighted and T2-weighted fat-suppressed sequences is accepted as a direct sign of a complete plantar plate rupture.^(14)^ In this study protocol, degenerative injury was most effectively identified in short axis intermediate-weighted images, in which it is possible to identify a small heterogeneous thickening or thinning of the inferolateral margin of the plantar plate. This view had a sensitivity of 92% for diagnosis of degenerative injury, with a negative predictive value of 95.8%.

Magnetic resonance imaging is the most accurate complementary exam for detecting plantar plate injuries,^(22)^ but they are small structures and are difficult to see. The observer in this study is highly trained and a specialist in the area, achieving a high degree of agreement for classification of degenerative injury cases and normal plantar plate. In clinical practice, for less well-trained observers, we believe that presence on T1-weighted fat saturation images of an area of thickening with intermediate signal, bordering the inferolateral margin of the metatarsal head (mild pericapsular fibrosis) and accompanied by a small intermediate signal interspersing the plantar plate or thickening or thinning of this structure on short axis intermediate-weighted sequences are strongly suggestive of a diagnosis of degenerative plantar plate injuries.

It is important to recognize that our study design was retrospective and cross-sectional, and no correlations were made with surgical or anatomopathological findings. However, the large sample, trained observer, and standardization of tests are the study’s strong points. Additional studies are needed that correlate these images with clinical course.

## CONCLUSION

In this study, degenerative injuries of the plantar plate were more often identified than complete plantar plate ruptures. The observer presented a high rate of intraobserver correlation, degenerative injuries were best identified on short axis intermediate-weighted images and with high sensibility of the direct signs of degenerative lesion (plantar plate thickening or thinning). The mild pericapsular fibrosis (below the intermetatarsal ligament) was the indirect sign with highest sensibility for the diagnosis of plantar plate degenerative lesion, and it was best evaluated on short axis T1-weighted images.
